# Identification of four new gene members of the KAP6 gene family in sheep

**DOI:** 10.1038/srep24074

**Published:** 2016-04-05

**Authors:** Huitong Zhou, Hua Gong, Jiqing Wang, Jolon M. Dyer, Yuzhu Luo, Jon G. H. Hickford

**Affiliations:** 1 Gansu Key Laboratory of Herbivorous Animal Biotechnology, Faculty of Animal Science and Technology, Gansu Agricultural University, Lanzhou 730070, China; 2 Department of Agricultural Science, Faculty of Agriculture and Life Sciences, Lincoln University, Lincoln 7647, New Zealand; 3 Lincoln Research Centre, AgResearch Limited, PO Box 8742, Lincoln 8140, New Zealand

## Abstract

KAP6 is a high glycine-tyrosine keratin-associated protein (HGT-KAP) family. This family is thought to contain multiple genes. In this study, we used a *KRTAP6* coding sequence to search the Ovine Genome (v3.1) and identified five homologous regions (R1–R5). All these regions contained an open reading frame, and they were either identical to, or highly similar to, sheep skin Expressed Sequence Tags (ESTs). Phylogenetic analysis revealed that R1–R5 were clustered with KAP6 sequences from different species and formed a group distinct to other HGT-KAPs. R1 was very similar to the characterised *KRTAP6-1* sequence, but the remaining genes appeared to be new. PCR primers were designed to amplify and confirm the presence of these new genes. Amplicons were obtained for all of the 96 sheep investigated. Six, five, three and six PCR-SSCP patterns representing six, five, three and six DNA sequences were observed for *KRTAP6-2* to *KRTAP6-5* respectively. *KRTAP6-2* and *KRTAP6-4* had five and three SNPs respectively. Three SNPs and a 45-bp insertion/deletion were detected for *KRTAP6-3*, and five SNPs and an 18-bp insertion/deletion were identified for *KRTAP6-5.* Allele frequencies for these KAP6 genes differed between Merino and Romney sheep.

Keratin-associated proteins (KAPs) are a key structural component of wool and hair fibres. They form a semi-rigid matrix in which the keratin intermediate filaments are embedded^1^ and hence they play an important role in determining fibre physico-mechanical properties. KAPs characteristically possess a high level of either cysteine, or both glycine and tyrosine, and based on their amino acid composition they can be allocated into three broad groups: the high sulphur (HS; ≤30 mol% cysteine), the ultra-high sulphur (UHS; >30 mol% cysteine) and the high glycine-tyrosine (HGT; 35–60 mol% glycine and tyrosine) KAPs^1^.

HGT-KAPs are predominantly present in the orthocortex of the wool fibre and are the first group of KAPs expressed after intermediate filament synthesis^2^. Wool varies considerably in HGT-KAP content ranging from less than 1% in Lincoln wool, to between 4% and 12% in Merino wool^3^. A reduction in the content of HGT-KAPs appears, at least in part, to be responsible for the felting lustre mutant found in Merino sheep^4^. The wide range in the proportional content of HGT-KAPs in different wools raises intriguing questions about the function of these proteins in the fibre.

Three HGT-KAPs families have been identified in sheep: KAP6, KAP7 and KAP8. In humans, the KAP7 and KAP8 families are found to contain one family member, whereas KAP6 has three members^5^. While earlier studies suggested single family members for KAP7 and KAP8 in sheep^6^, a second member of the KAP8 family has been recently identified^7^. RFLP-Southern hybridisation analysis suggested that mulitple KAP6 genes existed in sheep^8^, but the precise number of KAP6 gene family members has not been determined. To date only one gene (*KRTAP6-1*) has been characterised^8^, although the presence of three putative KAP6 genes has been suggested based on sequence analysis of amplified *KRTAP6*-like sequences^9^ and more recently Liu *et al*.^10^, also suggest the presence of more than one KAP6 gene.

Variation in a KAP6 gene has been reported to be associated with wool fibre diameter^11^, and recently a 57-bp deletion in *KRTAP6-1* has been found to be associated with variation in fibre diameter traits^12^. Given the potential effect of the KAP6 genes on the wool fibre characteristics, further study is needed to better understand the extent of diversity in the KAP6 gene family. The aim of this study was therefore to identify other KAP6 genes in the sheep genome and investigate the extent of variation in these genes.

## Results

### Identification of four newly identified KAP6 genes in the sheep genome

**Figure 1:**
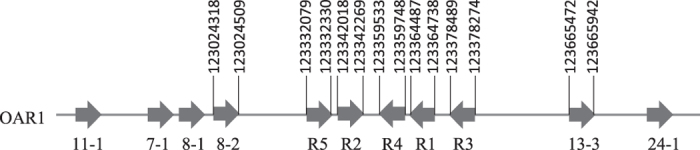
Location of sheep genome regions that were homologous to *KRTAP6-1*, together with six other previously identified *KRTAPs* on sheep chromosome 1. Horizontal arrow bars represent the coding regions of *KRTAPs* and the arrowheads indicate the direction of transcription. The numbers below the horizontal arrow bars indicate the name of the respective KAP gene (e.g., 11-1 represents *KRTAP11-1*). The five homologous regions are marked as R1 to R5. The nucleotide positions refer to NC_019458.

**Figure 2:**
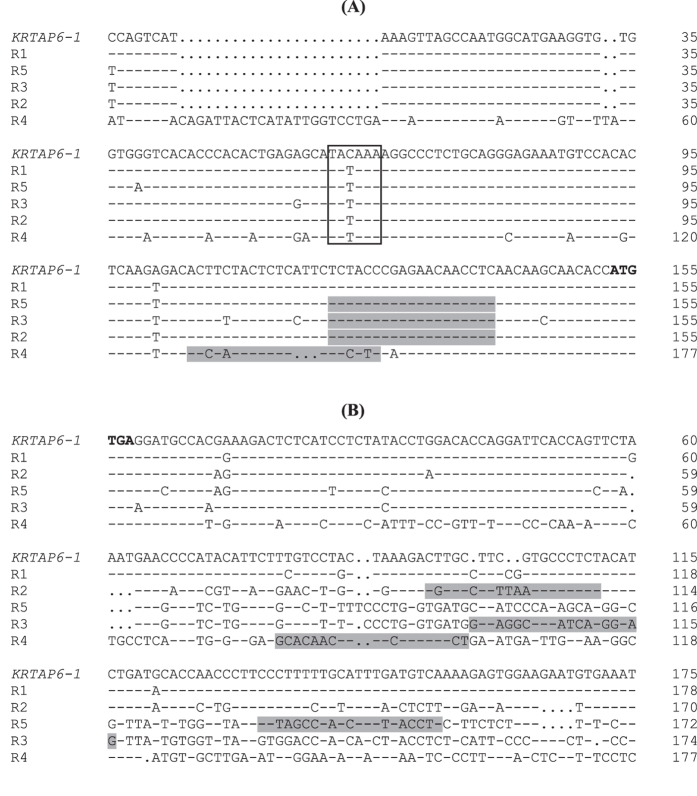
Comparison of the flanking sequences of the *KRTAP6-n* regions identified in this study. The 5′ flanking sequences (**A**) and the 3′ flanking sequences (**B**) of these regions (R1 to R5) were aligned with the published *KRTAP6-1* sequence. The translation initiation codon and stop codon are shown in bold. The putative TATA box sequences and transcription initiation sites are boxed and the primer binding regions for R2 to R5 are shaded. Dashes represent nucleotide sequences identical to the top sequence and dots are included as spacers to improve the alignments.

A BLAST search of the Ovine Genome Assembly v3.1 using the published *KRTAP6-1* coding sequence (GenBank M95719), revealed five homologous regions in the sheep genome (named R1 to R5). These homologous regions were clustered on sheep chromosome 1, in a region flanked by *KRTAP8-2* and *KRTAP13-3* (Fig. 1). R1 was very similar to the published *KRTAP6-1* sequence, with only one nucleotide difference within the coding sequence. This probably represents allelic sequence variation, or a sequencing error in one of these sequences. The remaining four regions (R2 to R5) showed sequence homology to the published *KRTAP6-1* sequence within the coding region, but the sequence similarity decreased outside the coding region, with major differences being observed in the 3′ flanking region (Fig. 2).

Each of R1 to R5 contained one open reading frame that would encode a protein that was glycine and tyrosine-rich. The open reading frames of R2, R4 and R5 were identical to a number of EST sequences in GenBank that were derived from sheep skin tissues, whereas the open reading frames of R1 and R3 were 99% and 95% homologous respectively across the entire reading frame to sheep skin Expressed Sequence Tags (ESTs) (Table 1).

**Figure 3:**
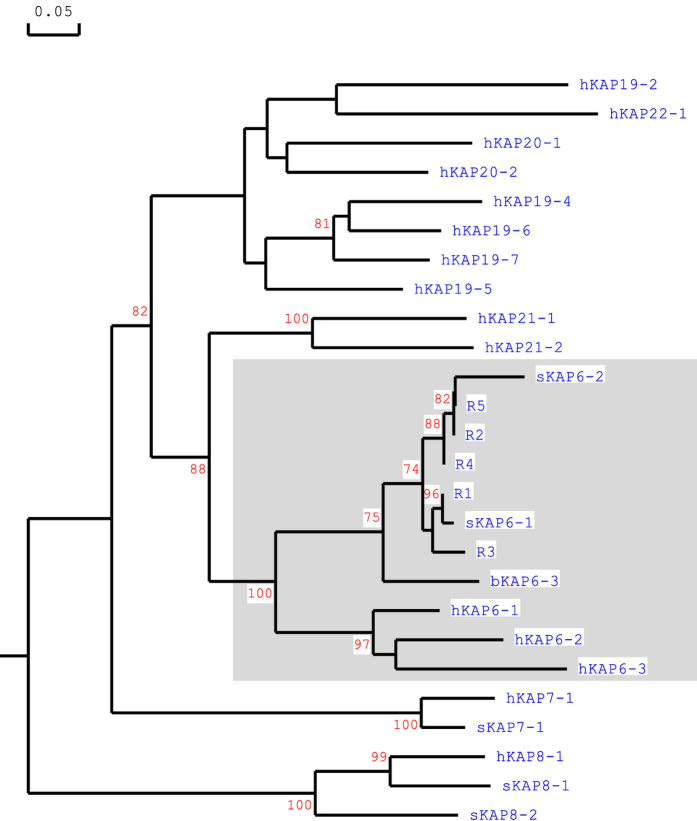
Phylogenetic tree of the sheep genomic regions identified, together with known HGT-KAP members from sheep, humans and cattle. The tree was constructed using the predicted amino acid sequences, except for sKAP6-2 for which a partial protein sequence was available. The ovine KAPs are indicated with the prefix “s”, the human KAPs have the prefix “h” and the bovine KAP6-3 sequence has the prefix “b”. The sequences clustered with the previously identified KAP6 sequences are shaded. The numbers at the forks indicate the bootstrap confidence values and only those equal to or higher than 70% are shown. The GenBank accession numbers for the human HGT-KAPs are AP001708 (hKAP6, hKAP19, hKAP20 and hKAP22), AP001709 (hKAP8 and hKAP21) and AB096962 (hKAP7). The GenBank accession numbers for the sheep HGT-KAPs are M95719, X05638, X05639 and KF220646 (for sKAP6-1, sKAP7-1, sKAP8-1 and sKAP8-2, respectively). The GenBank accession number for the cattle HGT-KAP is XM_883346 (bKAP6-3). The partial protein sequence of sKAP6-2 is sourced from Gillespie (1990).

The open reading frames identified from R1 to R5 were translated into amino acid sequences, and sequence comparison with known HGT-KAPs from sheep, humans and cattle revealed that these five homologous regions were clustered with other KAP6 sequences from different species and formed a group that is distinct to other HGT-KAP families (Fig. 3). It suggested the presence of five putative KAP6 genes in sheep. R1 was very similar to sheep KAP6-1 and probably represents the previously described ovine KAP6-1 gene^8^, whereas R2 to R5 represented four hitherto un-identified ovine KAP6 genes.

R2 and R5 were identical in their coding sequences and sequence differences were only observed in the 5′ and 3′ flanking sequences. While both the R2 and R5 regions showed high sequence homology to two previously reported *KRTAP6* sequences (KRTAP6*A and KRTAP6*C) that have been proposed to be derived from a putative *KRTAP6-2* 9; a slightly higher sequence similarity to these putative *KRTAP6-2* sequences was observed for R2. R3 exhibited a sequence pattern similar to a previously reported *KRTAP6* sequence (KRTAP6*E)^9^, derived from what Gong *et al*. described as a putative *KRTAP6-3*. R2 probably therefore represents *KRTAP6-2* and R3 probably represents *KRTAP6-3*. R4 and R5 would then represent two new *KRTAP6* members and could be named as *KRTAP6-4* and *KRTAP6-5* respectively.

In order to confirm whether these four un-identified KAP6 genes identified by a bioinformatic approach were present in sheep, four pairs of PCR primers were designed to specifically amplify parts of R2 to R5. An amplicon was obtained for each set of PCR primers in each sheep investigated.

### Variation in the four newly identified ovine KAP6 genes

**Figure 4:**
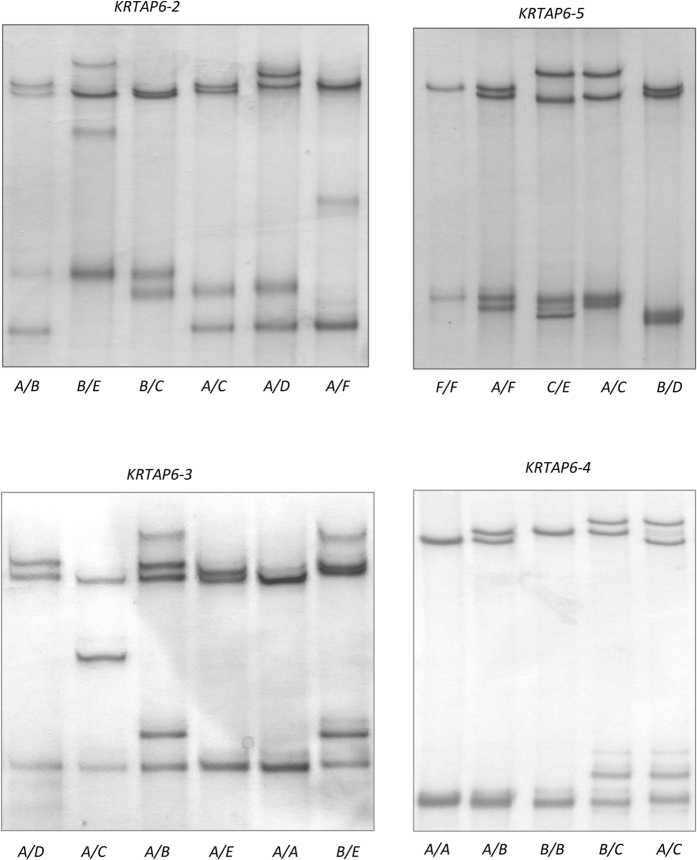
PCR-SSCP patterns of *KRTAP6-2, KRTAP6-3, KRTAP6-4* and *KRTAP6-5*. Sheep representative of either homozygous or heterozygous genotypes are shown.

**Figure 5:**
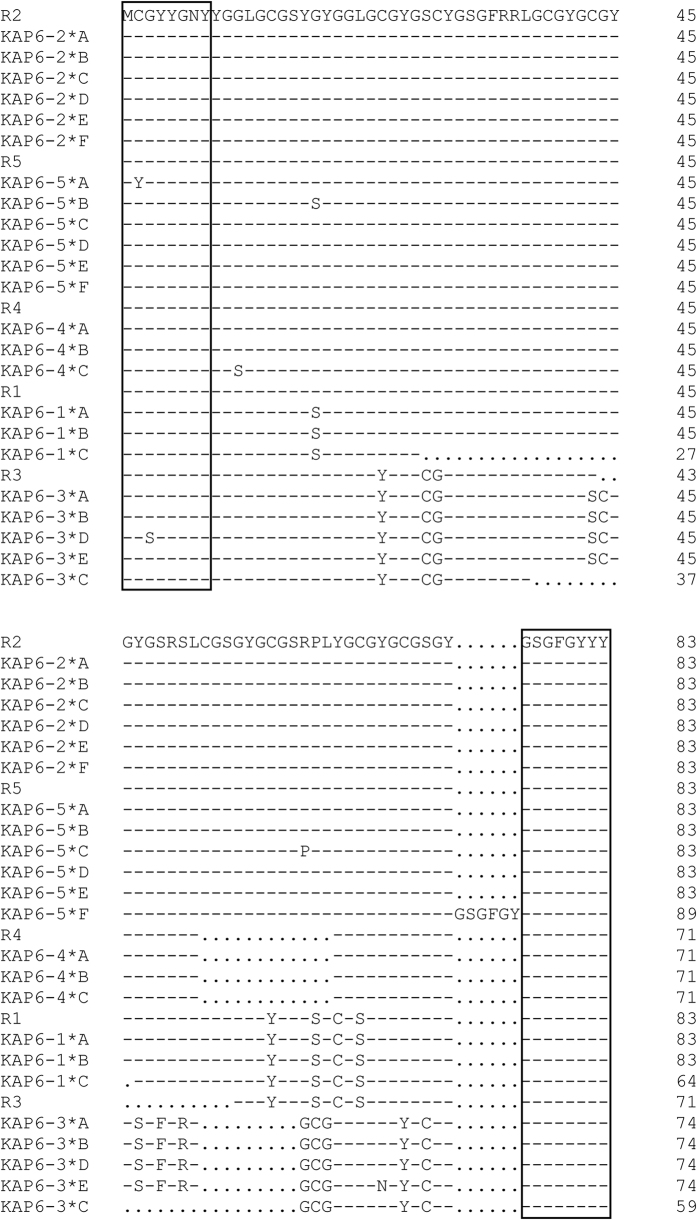
Alignment of the predicated amino acid sequences of the KAP6 sequences together with the homologous regions identified in the sheep genome. Dashes represent amino acid sequences identical to the top sequence, and dots have been introduced to improve the alignment. The sequence motifs unique to KAP6 proteins are shown in boxes.

The PCR amplicons from each region were analysed using SSCP analysis. For the R2 region, six PCR-SSCP banding patterns, representing six allele sequences, were observed (Fig. 4). Among these alleles, five SNPs were detected, and three of them were located within the coding region (Table 1). All the SNPs within the coding region were synonymous (Fig. 5). The sequence of allele *B* was identical to R2.

Five PCR-SSCP patterns representing five alleles were detected for region R3 (Fig. 4). In this region, there were three SNPs and a 45-bp insertion/deletion within the coding region. The 45-bp insertion/deletion was located in a region encoding a repeated amino acid sequence (Table 1 and Fig. 5). All but the *D* allele were identical to R3 except for the insertion/deletion, which was in the second half of the coding sequence.

Three PCR-SSCP patterns representing three allele sequences were observed for region R4 (Fig. 4). Three SNPs were identified and one SNP was located within the coding region (Table 1). This SNP was non-synonymous and would result in an amino acid change (Fig. 5). The sequence of allele *A* was identical to R4.

Six PCR-SSCP patterns representing six alleles were observed in region R5 (Fig. 4). Five SNPs were detected across these alleles and three of these were located in the coding region. Variation in the copy number of an 18-nucleotide repeat sequence was also found within the coding region (Table 1 and Fig. 5). Alleles *A* to *E* had one nucleotide sequence difference to R5, whereas allele *F* had a difference of an 18-nucleotide repeat sequence compared to R5.

### Comparison of allele frequencies between NZ Romney and Merino sheep

The frequencies of the *KRTAP6-2, KRTAP6-3, KRTAP6-4* and *KRTAP6-5* alleles in the NZ Romney and Merino sheep investigated in this study are shown in Table 1. Overall, there were more alleles found in the Merino sheep than in the NZ Romney, and differences in the common allele profiles between these two breeds were observed for *KRTAP6-3, KRTAP6-4* and *KRTAP6-5*. For *KRTAP6-3*, allele *B* was common in the Merino sheep, but it was not found in the NZ Romney sheep, whereas allele *D* was common in the NZ Romney sheep, but was absent in the Merino sheep. For *KRTAP6-4*, alleles *C* was rare in the Merinos, but was common in the NZ Romney sheep investigated. Likewise, allele *D* at *KRTAP6-5* was common in the Merino sheep, but was less commonly found in the NZ Romney sheep.

## Discussion

This study describes the identification of four new members of the KAP6 gene family. These genes are polymorphic with between three to six allelic variants found in the sheep studied. The identification of these KAP6 genes brings the number of KAP6 family members identified in sheep from one to five. This supports previous RFLP-hybridisation results that suggested the presence of multiple KAP6 genes in sheep^8^, and is in agreement with the recent observation of five putative KAP6 genes in the cattle genome^13^. It is consistent with Liu *et al*.*’s*^10^ finding of DNA sequences that appeared to be different to the known *KAP6-1* and *KAP6-2* sequences, but we chose not to use the sequences they report in our phylogenetic analysis. This decision was made because the approach Liu *et al*. used to produce their sequences involved the cloning of PCR amplicons into pGEM-T vectors before sequencing. This can lead to sequencing errors being made and this is reflected in their decision to treat ‘any mutation with a frequency of less than 1%’ as not being a SNP. Equally, the PCR primers use by Liu *et al*. to amplify *KAP6-n* sequences would not produce the claimed 498 bp amplicon, instead producing an amplicon based on the sequences they report of 117 bp.

The number of KAP6 genes reported here for sheep is higher than that reported for humans, where only three KAP6 genes have been identified^5^. The presence of more KAP genes in sheep than in humans has also been alluded to with the KAP8 family^7^. This suggests that sheep may have more KAP genes than humans, despite only a small number of ovine KAP genes having been described to date.

The five regions identified in this study exhibited high sequence similarity within the coding sequence, and two of them (R2 and R5) were identical. However, sequence similarity decreased in the flanking regions and a marked decrease in similarity was observed in the 3′ region. In mice, four KAP6 genes have been identified, and two of them encode an identical amino acid sequence, but possess differing 5′ and 3′ non-coding sequences^14^. High sequence similarity in coding regions, and low sequence similarity in the flanking regions, has also been described for ovine KAP1 genes^15^. The functional significance of this evolutionary pattern is unknown, but it is possible this may enable the production of more homogenous components of the fibre matrix, while also allowing for the independent regulation of expression of the individual genes.

Across species KAP genes have been divided into families based on sequence homology and in humans homology comparison has enabled 17 HGT-*KRTAP* sequences to be assigned into seven HGT-KAP families^5^. The five regions identified in this study were more closely related to KAP6 sequences from sheep, cattle and humans, than any other KAP sequence from any other KAP family (Fig. 3). This suggests that these homologous regions represent members of the extended KAP6 family, rather than different KAP families, a contention that is supported by the chromosomal location of these genes and other unique amino acid motifs (see Figs 1 and 4). The homologous regions identified were clustered in a chromosome region flanked by the KAP8 and KAP13 genes. This structure is similar to the KAP6 location reported in humans, but in humans the KAP22-1 gene is also found to be located among the KAP6 genes^5^. Despite the human homologue of KAP22-1 gene having not been identified in sheep to date, none of the regions identified in this study showed sequence homology to the human KAP22-1. Instead, all of these regions possess a conserved amino-terminal sequence (MCGYYNY) and a conserved carboxyl-terminal sequence (GSGFGYYY) that are unique to KAP6 proteins from other mammalian species^5^.

All the KAP6 genes identified in this study contained an open reading frame, and these open reading frames were either identical to, or showed high sequence homology with sheep skin EST sequences. This suggests that sheep have five functional and expressed KAP6 genes.

The finding of five KAP6 genes in sheep, along with the description of three KAP6 genes in humans^5^ and variable numbers of putative KAP6 genes found in other species^13^, suggests that KAP6 is a diverse family among species with the number of gene members varying considerably. For example, there appears to be an absence of any functional KAP6 gene in the hairless armadillo, to reports of nine KAP6 genes in rabbits and alpaca^13^. It is not known whether or how these differences are related to specific hair/wool features. However, the finding of the highest number of KAP6 genes in rabbits and alpaca, together with evidence of a linkage reported between KAP6 genes and wool fibre diameter in sheep^11^,^12^, suggests future investigation of KAP6 genes in sheep may further shed light on wool traits and their potential improvement through targeted breeding.

Despite the KAP6 genes being clustered and all of them exhibiting polymorphism, the extent and nature of the polymorphism found in these genes appear to vary. Firstly, a higher level of polymorphism was observed for *KRTAP6-2* and *KRTAP6-5*, whereas a lower level of polymorphism was observed for *KRTAP6-1* and *KRTAP6-4*. That is, there are six allele variants identified in each of *KRTAP6-2* and *KRTAP6-5*, but only three variants have been reported for *KRTAP6-4*, and three for *KRTAP6-1* in a previous study^11^. This appears to be consistent with the observation that *KRTAP6-2* and *KRTAP6-5* are closer to each other on the chromosome than the other *KRTAP6-n*’s and that *KRTAP6-1* and *KRTAP6-4* are closer to each other. The number of allelic variants identified in each KAP6 gene is consistent with the number of SNPs found in the gene.

Although more alleles may be found in each of these genes when more sheep from more breeds are analysed, the trends observed in the amount of polymorphism in each of these genes may stand.

The SNPs found in *KRTAP6-3* and *KRTAP6-5* are predominantly non-synonymous, whereas all the SNPs identified in *KRTAP6-1* and *KRTAP6-2* are synonymous. The nature of polymorphism is therefore not consistent with the physical location of the genes on the chromosome and it suggests that different mechanisms driving the accumulation of polymorphism may affect different genes.

A 45-bp deletion/insertion was detected for *KRTAP6-3*, and a 57-bp deletion/insertion has been reported previously for *KRTAP6-1*^11^. These deletions/insertions are located in the central region of the gene that contains repeated nucleotide and amino acid sequences. While a deletion/insertion was also observed for *KRTAP6-5*, it is relatively small (18-bp) and is located in the 3′ region of the gene. This 18-bp deletion/insertion results in a variation in the number of the hexa-peptide repeats in the carboxyl-region of the KAP6-5 protein. Length variation was not observed for *KRTAP6-2* and *KRTAP6-4*.

The identification of two allele sequences (KRTAP6-2*B and KRTAP6-4*A) that are identical to R2 and R4 confirms that the R2 and R4 sequences reported in the Ovine Genome Assembly v3.1 are genuine. The high sequence homology between R5 and *KRTAP6-5* allele sequences identified in this study and between R1 and previously described *KRTAP6-1* sequences^11^, suggests that the R1 and R5 sequences are either genuine as the sequence differences observed probably reflect new allelic variation, or contain sequencing errors. A sequence identical or highly homologous to R3 was not identified in the sheep investigated. However, R3 appears to be a recombinant sequence of *KRTAP6-1* and *KRTAP6-3*, suggesting that the R3 sequence reported in the Ovine Genome Assembly v3.1 may not be genuine, and may have come about because of technical errors in assembling the sequences.

It is interesting to note that the Merino sheep tended to have more alleles of the KAP6 genes than the NZ Romney sheep, and that some alleles that were common in one breed were found to be absent or rare in the other breed. This suggests that KAP6 genes may play a role in regulating wool traits, or can be used as gene-marker for wool traits, but caution is needed in claiming this as the sheep samples investigated may not be representative of the breeds. Further investigation on more sheep that have wool trait data recorded is needed to confirm this finding.

## Materials and Methods

All research involving animals were carried out in accordence with the Animal Welfare Act 1999 (New Zealand Govertment) and the collection of sheep blood drops by nicking sheep ears is covered by Section 7.5 Animal Identification, of the Animal Welfare (Sheep and Beef Cattle) Code of Welfare 2010; a code of welfare issued under the Animal Welfare Act 1999 (New Zealand Government).

### Sheep investigated and DNA isolation

Ninety-six sheep from the Merino breed (n = 48, sourced from four farms) and New Zealand (NZ) Romney breed (n = 48, sourced from four farms), were investigated. Blood samples were collected onto FTA cards (Whatman BioScience, Middlesex, UK) and genomic DNA was purified using a two-step washing procedure described in Zhou *et al*.^16^.

### Bioinformatic analysis of the sheep genome sequence

The coding sequence of the published ovine *KRTAP6-1* sequence (GenBank M95719) was used in a BLAST search of the Ovine Genome Sequence Assembly v3.1. Genome sequence segments that showed high homology (E value < e^−30^) with this coding sequence were regarded as putative KAP6 gene family members, and the open reading frames were identified.

### PCR primers and amplification of sheep genomic DNA

Sequences flanking the open reading frames of the putative KAP6 gene family members (*KRTAP6-n*) identified above, were used to design PCR primers for amplifying these homologous regions individually from sheep genomic DNA; with the exception for *KRTAP6-1* which has been characterized previously^8^,^11^. The PCR primer sequences used are shown in Table 2. All the primers were synthesized by Integrated DNA Technologies (Coralville, IA, USA).

PCR amplification was performed in a 15-μL reaction containing the genomic DNA on one 1.2-mm punch of FTA paper, 0.25 μM of each primer, 150 μM of each dNTP (Bioline, London, UK), 2.5 mM of Mg^2+^, 0.5 U of Taq DNA polymerase (Qiagen, Hilden, Germany) and 1× reaction buffer supplied with the enzyme. The thermal profile consisted of 2 min at 94 °C, followed by 35 cycles of 30 s at 94 °C, 30 s at the annealing temperatures shown in Table 1 and 30 s at 72 °C, with a final extension of 5 min at 72 °C. Amplification was carried out in S1000 thermal cyclers (Bio-Rad, Hercules, CA, USA).

Amplicons were visualized by electrophoresis in 1% agarose gels (Quantum Scientific, Queensland, Australia), using 1× TBE buffer (89 mM Tris, 89 mM boric acid, 2 mM Na_2_EDTA) containing 200 ng/mL of ethidium bromide.

### Screening for variation in *KRTAP6-n*

PCR amplicons were screened for sequence variation using SSCP analysis. A 0.7-μL aliquot of each amplicon was mixed with 7 μL of loading dye (98% formamide, 10 mM EDTA, 0.025% bromophenol blue, 0.025% xylene-cyanol). After denaturation at 95 °C for 5 min, the samples were rapidly cooled on wet ice and then loaded on 16 cm × 18 cm, 10% acrylamide:bisacrylamide (37.5:1) (Bio-Rad) gels. Electrophoresis was performed using Protean II xi cells (Bio-Rad) in 0.5× TBE buffer, under the electrophoretic conditions shown in Table 2. Gels were silver-stained according to the method of Byun *et al*.^17^.

### Sequencing of allelic variants and sequence analysis

PCR amplicons representing different banding patterns from sheep that appeared to be homozygous were sequenced in both directions at Lincoln University, New Zealand. Alleles that were only found in heterozygous sheep were sequenced using a rapid approach described previously^9^. Briefly, a band corresponding to the allele was excised as a gel slice from the polyacrylamide gel, macerated, and then used as a template for re-amplification with the original primers. This second amplicon was then sequenced.

Sequence alignments, translations and phylogenetic analysis were carried out using DNAMAN (version 5.2.10, Lynnon BioSoft, Vaudreuil, Canada).

## Additional Information

**How to cite this article**: Zhou, H. *et al*. Identification of four new gene members of the KAP6 gene family in sheep. *Sci. Rep.* **6**, 24074; doi: 10.1038/srep24074 (2016).

## Figures and Tables

**Table 1:**
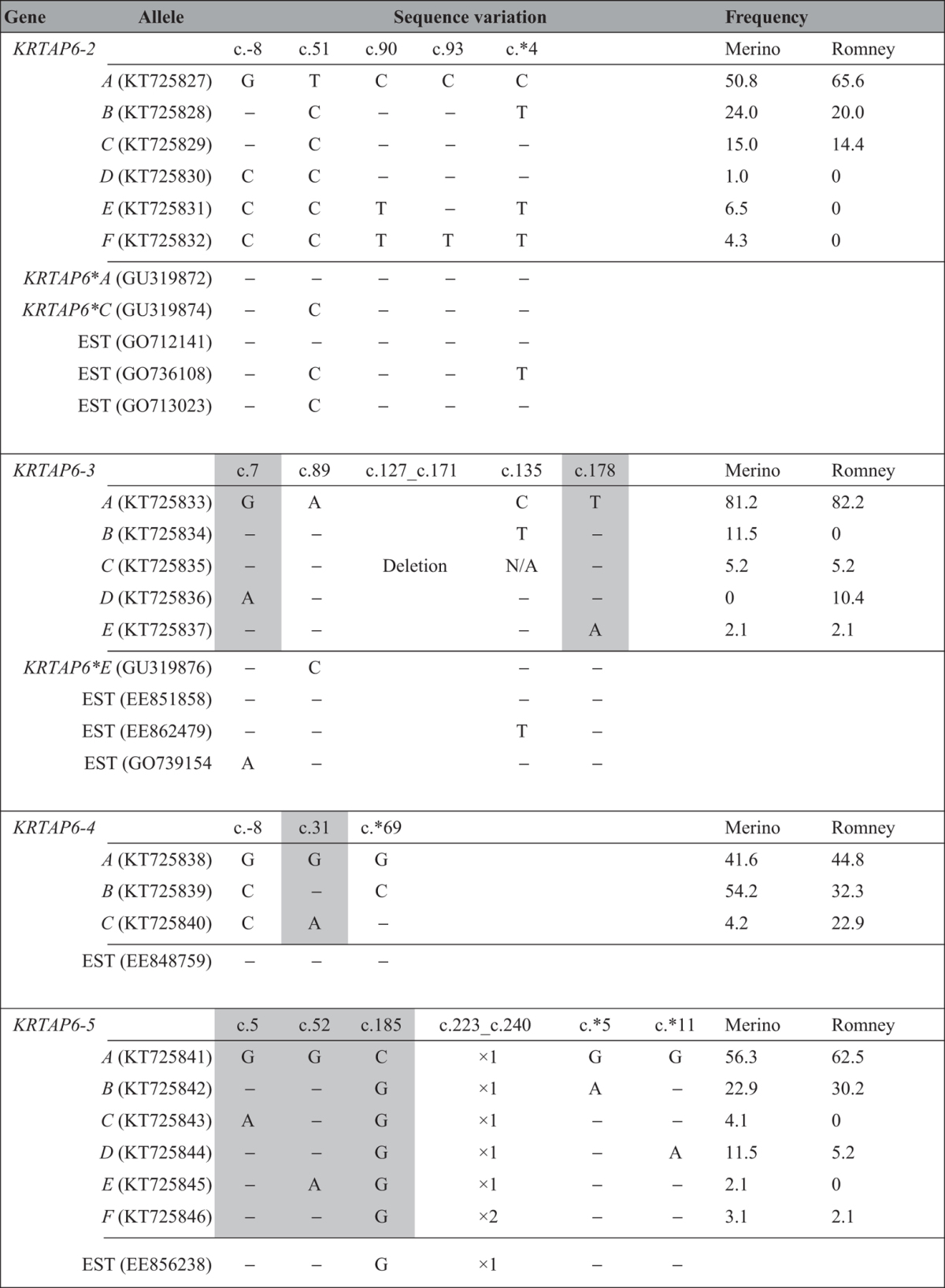
Sequence variation, comparison and allele frequencies of *KRTAP6-2* to *KRTAP6-5*.

Sequences from putative *KRTAP6-2* and *KRTAP6-3*, together with the sheep skin EST sequences that are either identical or highly homologous to the allele sequences reported here, are compared. GenBank accession numbers are shown in brackets. Non-synonymous SNPs are shaded. The reference sequences for *KRTAP6-2, KRTAP6-3, KRTAP6-4* and *KRTAP6-5* are KT725827, KT725833, KT725838 and KT725841, respectively. Dashes represent nucleotides identical to the top sequences of the corresponding genes.

**Table 2:** PCR primers used for amplifying *KRTAP6-n.*

Gene	PCR primer (5′-3′)	Expected size	Annealing temp.	SSCP conditions
*KRTAP6-2*	KAP6-up1: TCTACCCGAGAACAACCTC	429 bp	60 °C	10%, 9 °C, 320 V, 18 h
	KAP6-dn2: AGAGGGCATTAAAAGGCACG			
*KRTAP6-3*	KAP6-up1: TCTACCCGAGAACAACCTC	399 bp	60 °C	9%, 3 °C, 350 V, 18 h
	KAP6-dn3: CTTCCATGATGCAGCCTAAC			
*KRTAP6-4*	KAP6-up2: CACCTATACTCTCCTCCATC	358 bp	60 °C	10%, 7 °C, 320 V, 18 h
	KAP6-dn4: AGAAGTCTGTAGTGTTGTGC			
*KRTAP6-5*	KAP6-up1: TCTACCCGAGAACAACCTC	473 bp	60 °C	10%, 12 °C, 300 V, 18 h
	KAP6-dn5: TAGGTTAAAAGGTAGGCTAGG			
